# Optimizing mangrove afforestation: Mollusc biodiversity comparisons reveal optimal mudflat–mangrove area ratio

**DOI:** 10.1002/ece3.70330

**Published:** 2024-09-22

**Authors:** Yufeng Lin, Zifeng Luo, Xuan Gu, Yijuan Deng, Pingping Guo, Guogui Chen, Wenqing Wang, Mao Wang

**Affiliations:** ^1^ Key Laboratory of the Ministry of Education for Coastal and Wetland Ecosystems, College of the Environment & Ecology Xiamen University Xiamen China; ^2^ Zhangjiang Estuary Mangrove Wetland Ecosystem Station, National Observation and Research Station for the Taiwan Strait Marine Ecosystem Xiamen University Zhangzhou China; ^3^ Engineering Research Center of Fujian Province for Coastal Wetland Protection and Ecological Recovery, College of the Environment & Ecology Xiamen University Xiamen China

**Keywords:** ecological restoration, functional diversity, mangrove, mollusc, mudflat

## Abstract

In recent decades, mangrove wetlands globally have suffered from human activities and climate change, leading to issues like area reduction, degraded ecological functions and declining biodiversity. Restoration efforts, primarily through mangrove afforestation (i.e. mangrove plantation in mudflats), have been widespread, yet they often overlook the significance of unvegetated mudflats. In addition, under the condition that the total area of suitable mudflats is limited, the problem of what is the threshold of mangrove forests and unvegetated mudflats to better protect mangrove biodiversity has not been solved. Therefore, this study conducted a field survey of molluscs in mangrove wetlands in Hainan Island in China and explored the relative importance of mangroves and unvegetated mudflats through taxonomic alpha diversity and functional diversity. The results showed that (1) mollusc abundance of unvegetated mudflats was notably higher than this of mangrove forests, and the species richness, functional richness and functional vulnerability were significantly lower than those of mangrove forests; (2) the abundance and functional vulnerability of molluscs were mainly affected by sediment properties (pH, interstitial water salinity, median diameter, total nitrogen, C/N ratio), while the species richness and functional richness of molluscs were primarily influenced by vegetation structure (plant density); and (3) retaining at least 20% of the unvegetated mudflat area could well protect the biodiversity of mangrove wetlands. To our knowledge, our study is the first to propose the proportion of mangrove forests and unvegetated mudflats on the basis of benthic biodiversity, providing theoretical support and decision‐making reference for mangrove protection and restoration.

## Graphical abstract

The area of mangrove forest has been increased by mudflat afforestation worldwide, yet the problem of how to allocate the mudflat–mangrove area has been neglected. Mudflats had higher molluscs abundance and lower functional vulnerability, which were mainly affected by sediment properties, while mangroves hosted greater taxonomic and functional richness, which were primarily influenced by vegetation structure. Retaining at least 20% of mudflat area was crucial for mangrove wetland biodiversity conservation.

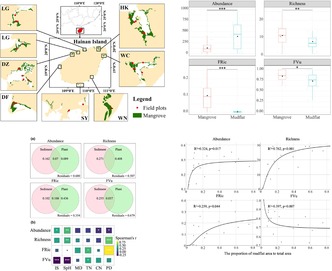

## 1 INTRODUCTION

Over the past 50 years, global mangrove coverage has diminished by over one‐third, a trend comparable to that observed in tropical rainforests (Duke et al., 2007). In response to this concerning loss, initiatives for mangrove ecological restoration have been initiated worldwide, primarily focusing on the afforestation of mudflats (Primavera et al., 2011). Lewis (2005) summarized the primary factors contributing to the inadequacies observed in mangrove afforestation endeavours, believing that ecological restoration efforts in mangrove wetlands have often been erroneously equated with mere tree planting, with afforestation activities on unvegetated mudflats being a central contributor to these failures. Notably, it is essential to distinguish between mangroves and mangrove ecosystems. The latter comprises four fundamental geomorphic units: mangrove forests, mudflats, shallow water areas and tidal creeks (Fu, Tang, et al., 2021). Fan et al. (2022) showed that the baseline value of wetlands outside the forest (including mudflat, shallow water area and tidal creek) attributed to mangroves in China was as high as 7.3283 million Yuan hm^−2^ a^−1^. Each unit is indispensable for the maintenance of the structure and function of the mangrove wetland ecosystem. Consequently, an excessive focus on restoring a single geomorphic unit, such as vegetation, hampers the comprehensive restoration and functioning of ecological processes at the ecosystem level, thereby constraining the efficacy of ecological restoration initiatives (Lee et al., 2019). Yet, determining the optimal allocation of area proportions for each geomorphic unit, particularly mangrove forests and mudflats, to achieve better ecological conservation benefits remains a critical knowledge gap (Yang et al., 2021).

Benthic organisms serve as pivotal indicators of the ecological conservation benefits derived from coastal wetlands. Within mangrove ecosystems, distinctive benthic communities thrive (Cannicci et al., 2008; Nagelkerken et al., 2008), comprising a diverse array of sessile and mobile macrobenthos, notably molluscs (Cannicci et al., 2009; Geist et al., 2012), which exhibit remarkable levels of abundance and biomass (Lee, 2008). Both gastropods and bivalves function as significant bioengineers, shaping the biochemical properties of mangrove sediments and water (Cannicci et al., 2008). Molluscs, in particular, serve as essential intermediaries within the coastal food web, linking primary detritus at the sediment base to higher trophic level consumers (Kristensen, 2008; Lee, 2008), thereby facilitating a bottom‐up influence within detrital food chains (Chen et al., 2023). Owing to their limited mobility and sensitivity to environmental fluctuations, macrobenthos also serve as crucial environmental indicators (Cannicci et al., 2009), with significant economic value (Lee, 2008) and potential for carbon sequestration (Donato et al., 2011).

Mangroves and unvegetated mudflats are two types of intertidal habitats that are arranged together and support each other (Jennerjahn, 2020; Meijer et al., 2021). Macrobenthos move between these two habitats. The complex root structure of mangrove vegetation not only provides suitable habitat but also results in higher organic content in the sediments due to mangrove detritus, which serves as a food source, leading to differences in macrobenthos between the two habitats (Chen et al., 2013; Nozarpour et al., 2024). Additionally, mangrove vegetation alters the properties of the sediment within the forest, such as salinity and acidity (Gleason et al., 2003; Lee & Shih, 2004). These sedimentary factors were important in influencing the composition and distribution of macrobenthos. Numerous investigations have delved into the disparities of macrozoobenthos populations between mangrove forests and mudflats at the local scale (Chen et al., 2007; Ebadzadeh et al., 2024; Pan et al., 2021), and it is controversial. Mounting evidence underscores the pivotal role of diverse macrobenthic organisms in mangrove functioning (Cannicci et al., 2008; Lee, 2008). Although some studies have focused on the diversity and taxonomic trait of assemblages at the local scale (Leung, 2015; Leung & Cheung, 2017; Nozarpour et al., 2024), functional diversity and vulnerability deserve further study. Both are indispensable for evaluating the ecosystem's capacity to furnish vital services and gauge the repercussions of disturbances on ecosystem integrity (Cannicci et al., 2021; Fischer et al., 2021). Nevertheless, there exists a lack of studies elucidating the functional diversity and vulnerability of molluscs across distinct geomorphological units at regional scales, particularly within mangrove forests and mudflats. Such knowledge gaps impede the formulation and execution of policies aimed at biodiversity conservation and climate change mitigation amidst mangrove restoration efforts.

Environmental heterogeneity (including geomorphic unit diversity) is widely recognized as a factor in maintaining biodiversity (Thomsen et al., 2022; Whalen et al., 2016). Higher levels of environmental heterogeneity imply the presence of more microhabitats and microclimatic conditions, thus accommodating a greater variety of species with different ecological niches, which facilitates the coexistence of more species (Hillebrand, 2004; Schlacher et al., 2007; Williams et al., 2010). The complex community structures within mangrove forests offer refuge to fauna, mitigating predation risks (Nagelkerken et al., 2008; Peng et al., 2017). For example, molluscs of Littorinidae with diverse shells tend to inhabit substrates with analogous colours to evade predation (Parsonage & Hughes, 2002). Moreover, mangroves provide nutrients for animal growth and promotes energy flow and species turnover with adjacent ecosystems, such as coral reefs and sea grass beds (Kathiresan, 2014). Notably, not only mangrove forest but also unvegetated mudflats are important for supporting biodiversity and ecosystem services (Dissanayake et al., 2018). Mudflats harbour abundant benthic fauna, serving as a livelihood for local communities (Sheaves et al., 2016) and a crucial food source for birds and fish (Choi et al., 2022; Marley et al., 2020; Studds et al., 2017). Even if it succeeds in transforming all the mudflats outside the mangrove forests into mangroves, it will encroach upon the foraging grounds of waterbirds, which is equivalent to wiping out the ‘rice bowl’ of waterbirds (Primavera et al., 2011; Primavera & Esteban, 2008). Moreover, mudflats exhibit significant carbon sequestration potential akin to that of coastal vegetated ecosystems, particularly in estuarine settings where hydrodynamic conditions facilitate carbon burial and organic matter influx from river sediments (Chen, Wang, Li, et al., 2020; Choi & Wang, 2004; Roberts et al., 2015). Therefore, we should follow the principle of ‘afforestation where it is suitable for afforestation, and preserve the mudflat where it is suitable for preserving mudflat’. But there is a lack of field survey data to verify the reserve benchmark of unvegetated mudflat area for mangrove afforestation. It is urgent to determine the ratio of mangrove forest to unvegetated mudflat to support the subsequent protection and restoration of mangrove wetland ecosystem.

This study conducted a field survey of molluscs in mangrove forests and unvegetated mudflats at eight representative sites in Hainan Island, which is the second largest island in China and has the most species of mangroves with 26 true mangroves and 11 semi‐mangroves (Bai et al., 2021). The aim of our investigation was to elucidate the relative importance of different geomorphic units (including mangrove forest and unvegetated mudflats) in the mangrove wetland ecosystem through two aspects: taxonomic alpha diversity and functional diversity and to explore the optimal ratio of mudflat–mangrove area. We sought to address three scientific questions: (1) Are there significant differences in taxonomic alpha diversity, functional diversity of molluscs between mangrove and unvegetated mudflats? (2) What are the key biotic and abiotic factors that affect their differences? (3) What is the ratio of mangrove and unvegetated mudflat area to better protect mangrove biodiversity?

## 2 MATERIALS AND METHODS

### 2.1 Study area

Our study was conducted on Hainan Island (18°10′ N–20°10′ N, 108°37′ E–111°03′ E), which located in the northwest of the South China Sea. Hainan Island has a tropical monsoon climate with an average annual temperature of 24.2°C (Meng et al., 2022). The tide types vary from place to place, with a mean tide height of about 2 m. Annual precipitation ranges from 1000 to 2600 mm, with an average annual precipitation of 1639 mm. The rainy season is from May to October each year, with a total precipitation of about 1500 mm, accounting for 70%–90% of the total annual precipitation. The rainy season is from November to April of the next year. There are numerous bays, estuaries and long coastlines in Hainan Island, which provide superior conditions for the growth and reproduction of mangroves. The area of mangroves is 4710 ha (Fu, Tang, et al., 2021), which are mainly distributed in Dongzhaigang, Qinglangang, Sanyagang and Xinyinggang. Dongzhaigang National Nature Reserve is the first mangrove reserve area in China. Hainan Island has the highest biodiversity of all the mangroves in China. There are 37 species of mangroves in China, among which Hainan Island has the largest number of mangroves, with 26 true mangroves and 11 semi‐mangroves (Bai et al., 2021). Not only that, the island has the highest carbon storage capacity of any mangrove in China (Liu et al., 2014). Therefore, Hainan Island is considered a hot spot for mangrove research and conservation (Chen, Gu, et al., 2021).

### 2.2 Sampling and biotic and abiotic factors

**FIGURE 1:**
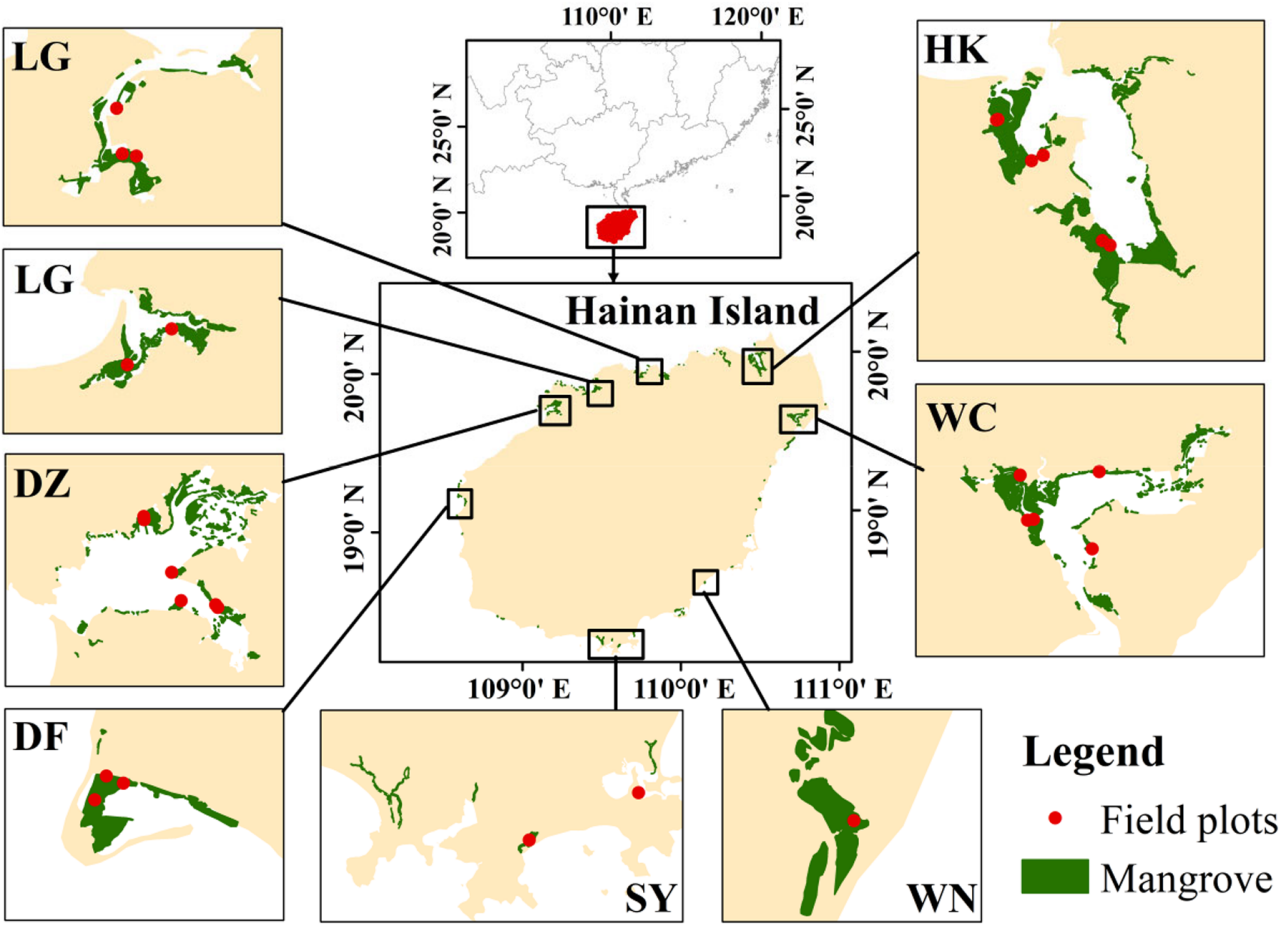
Distribution and field sampling sites of mangroves in Hainan Island, China (DF, Dongfang; DZ, Danzhou; HK, Haikou; LG, Lingao; SY, Sanya; WC, Wenchang; and WN, Wanning).

According to the distribution characteristics of mangroves in Hainan Island, a total of 56 plots were selected from 8 representative sites and 28 transects (Figure 1 and Table 1). The mangrove biodiversity and its environmental factors were investigated in the rainy and dry seasons of the east and west coasts from November 2021 to October 2022. Additional information on each study site is presented in Table 1.

**TABLE 1:** Detailed description of field sampling sites in Hainan Island, China.

Region	Site	Transect	Longitude (E)	Latitude (N)	Number of mangrove species
East coast	HK (Haikou)	HK1	110.5408	19.9970	8
		HK2	110.5415	19.9973	6
		HK3	110.5999	19.9318	13
		HK4	110.5960	19.9342	13
		HK5	110.5652	19.9787	5
		HK6	110.5591	19.9758	7
	SY (Sanya)	SY1	109.6158	18.2258	10
		SY2	109.7023	18.2632	12
	WC (Wenchang)	WC1	110.7974	19.6018	12
		WC2	110.8004	19.6023	13
		WC3	110.7935	19.6252	12
		WC4	110.8345	19.6271	15
		WC5	110.8309	19.5870	11
	WN (Wanning)	WN1	110.1793	18.5971	9
West coast	DF (Dongfang)	DF1	108.6340	19.2168	2
		DF2	108.6367	19.2157	2
		DF3	108.6321	19.2130	1
	DZ (Danzhou)	DZ1	109.2566	19.7692	5
		DZ2	109.2568	19.7676	6
		DZ3	109.2702	19.7420	5
		DZ4	109.2927	19.7249	5
		DZ5	109.2917	19.7262	3
		DZ6	109.2748	19.7282	4
	LG (Lingao)	LG1	109.8439	19.9206	8
		LG2	109.8408	19.9211	8
		LG3	109.8396	19.9310	4
		LG4	109.5539	19.8570	4
		LG5	109.5367	19.8430	7

For molluscs sampling, we set up 1–6 transects of 200–1500 m in length at each site, with an interval of at least 100 m between transects. Two sampling plots (including mangrove forest and mudflat) were set up in each transect. Five 25 cm × 25 cm quadrants were randomly selected from each sampling plot, with a depth of 30 cm to collect sediment samples. Each mud sample was panned with a 1‐mm mesh sieve (Ma et al., 2020). Then, the mollusc samples were selected and fixed in 70% alcohol and brought back to the laboratory for classification and identification (Liu et al., 2016).

During the sampling period, the following environmental variables were measured in situ after biological samples were collected in each quadrat. Interstitial water salinity (IS) was measured using a portable salinometer (PAL‐SALT Mohr, ATAGO, Japan) in each quadrat for three replicates. Latitude and longitude (coordinates) were determined by a hand‐held GPS. Mangrove wetland area was obtained from National Earth System Science Data Center, National Science & Technology Infrastructure of China (http://www.geodata.cn).

After removing surface humus at each sampling plot, three quadrats were randomly selected to collect about 150 g of 0–8 cm topsoil, which was brought back to the laboratory to air dry. And then the sediment moisture content (SMC) was calculated. The formula is as follows:

$$\mathrm{SMC}=\frac{W_{1}-W_{2}}{W_{2}\;}\times 100\%$$

where *W*_*1*_ is the weight of wet sediment and *W*_*2*_ is the weight of dried sediment.

Air‐dried sediment samples were removed of impurities and pass through a 20‐mesh sieve for subsequent processing. Sediment pH (SpH) was prepared according to the soil–water ratio of 2.5:1, and the pH of the extract was measured by a pH meter (Thunder magnetic pHS‐3C, China). The median diameter (MD) of the sediment was measured using a laser particle size analyser (Malvern 2000, UK). The remaining sediment samples were passed through a 100‐mesh sieve. Sediment organic carbon (TOC) and organic matter (TOM) were determined by potassium dichromate volumetric method. Sediment total nitrogen (TN) was determined by Kjeldahl method. The C/N ratio (CN) was then obtained by dividing sediment organic carbon by sediment total nitrogen.

Biotic factors are defined as the number of mangrove species (MS), mean tree height (H), mean diameter at breast height (DBH) and plant density (PD). The number of mangrove species was based on field survey records. From July to August 2023, three 5 m × 5 m plant quadrats were set in each plot (except mudflats). The DBH and tree height of all trees were measured. The DBH was generally 130 cm for trees and 30 cm for shrubs (Bai et al., 2021). Plant density was calculated as the number of individuals per quadrat divided by the area of the quadrat.

### 2.3 Statistical analyses

In this study, the abundance of molluscs is the number of individuals recorded at each sampling site. The abundance was log‐transformed. Species richness was used to quantify the alpha diversity of mollusc communities (Tuomisto, 2010). We selected five groups of functional traits, namely individual size, lifestyle, tree‐climbing behavior, vertical range of motion and feeding pattern, which basically represent the characteristics known to affect the ecosystem functions of mollusc communities, such as space utilization capacity, material circulation, energy flow and response to environmental changes (Bremner, 2008; Leung & Cheung, 2017; Van Der Linden et al., 2017). We calculated three functional diversity indices using ‘mFD’ package (Magneville et al., 2022): functional richness (FRic), functional vulnerability (FVu) and community‐weighted mean (CWM). FRic reflects the range of traits of organisms in a community. A high FRic indicates a large number of trait combinations, whereas a low FRic indicates the presence of only a few traits in the community. CWM is defined as the weighted average of the relative abundance of each species in a community, which is of great significance for evaluating community dynamics and ecosystem processes (De Bello et al., 2021). The formulas for FVu are calculated following Mouillot et al. (2014), as follows:

$$\mathrm{FVu}=\frac{\sum_{i=1}^{\mathrm{FE}}\min\left(n_{i}=1\right)}{\mathrm{FE}}$$

where FE is the total functional groups at a site and *n*_*i*_ is the number of species in functional entity *i*. Because the assumption of normality of biodiversity was not met, the nonparametric Wilcoxon signed‐rank test (Ren et al., 2023) was used to test for differences in mollusc biodiversity between unvegetated mudflats and mangroves.

Spearman correlation analysis was used to explore the strength of the association between any two environment variables. Highly correlated environmental factors were eliminated, and variables with the lowest correlation (Spearman's rho < 0.7) were selected to be retained. To explain the importance of each set of variables, a variation partitioning analysis (VPA) was performed. VPA uses *R*_adj_^2^ to estimate unbiased interpretation rates of each variable combination (pure sediment factor, pure plant factor), which is a common method in biogeography research. The *p*‐value of each combination is tested by a series of partial RDA models. In order to investigate the impact of remaining environmental factors on these biodiversity indicators, we calculated the Spearman correlation coefficient and its significance. All analyses in this part were conducted using ‘vegan’ and ‘stats’ in R v.4.1.2.

In order to explore the curve fitting model of the ratio of mudflat area to total area [*x*] and mollusc biodiversity [*f*(*x*)], the following mathematical fitting model was established:

The linear function

$$f\left(x\right)=\mathrm{ax}+b$$

The logarithmic function

$$f\left(x\right)=\log_{a}x+b$$

The reciprocal function

fx=a1x+b

The quadratic function

$$f\left(x\right)=ax^{2}+\mathrm{bx}+c$$

The cubic function

$$f\left(x\right)=ax^{3}+bx^{2}+\mathrm{cx}+d$$

The compound function

$$f\left(x\right)={\left(\mathrm{ab}\right)}^{x}$$

The power function

$$f\left(x\right)=ax^{b}$$

The S function

fx=11+exp−x

The increasing function

$$f\left(x\right)=\exp\left(a+\mathrm{bx}\right)$$

The exponential function

$$f\left(x\right)=a^{x}$$

The evaluation of each function involved considering the *R* ^2^ coefficient, standard error (SSE) and regression significance. Subsequently, the model demonstrating the best fit and the strongest correlation was chosen. This part was conducted using SPSS25.0.

## 3 RESULTS

A total of 88 mollusc taxa of 37 families were found in Hainan Island (Figure S1). Among them, the most species were gastropoda, accounting for 61.4%. Ellobiidae, Veneridae, Potamididae and Littorinidae were the dominant groups (Figure S1).

### 3.1 Differences in mollusc biodiversity between unvegetated mudflat and mangrove forest

**FIGURE 2:**
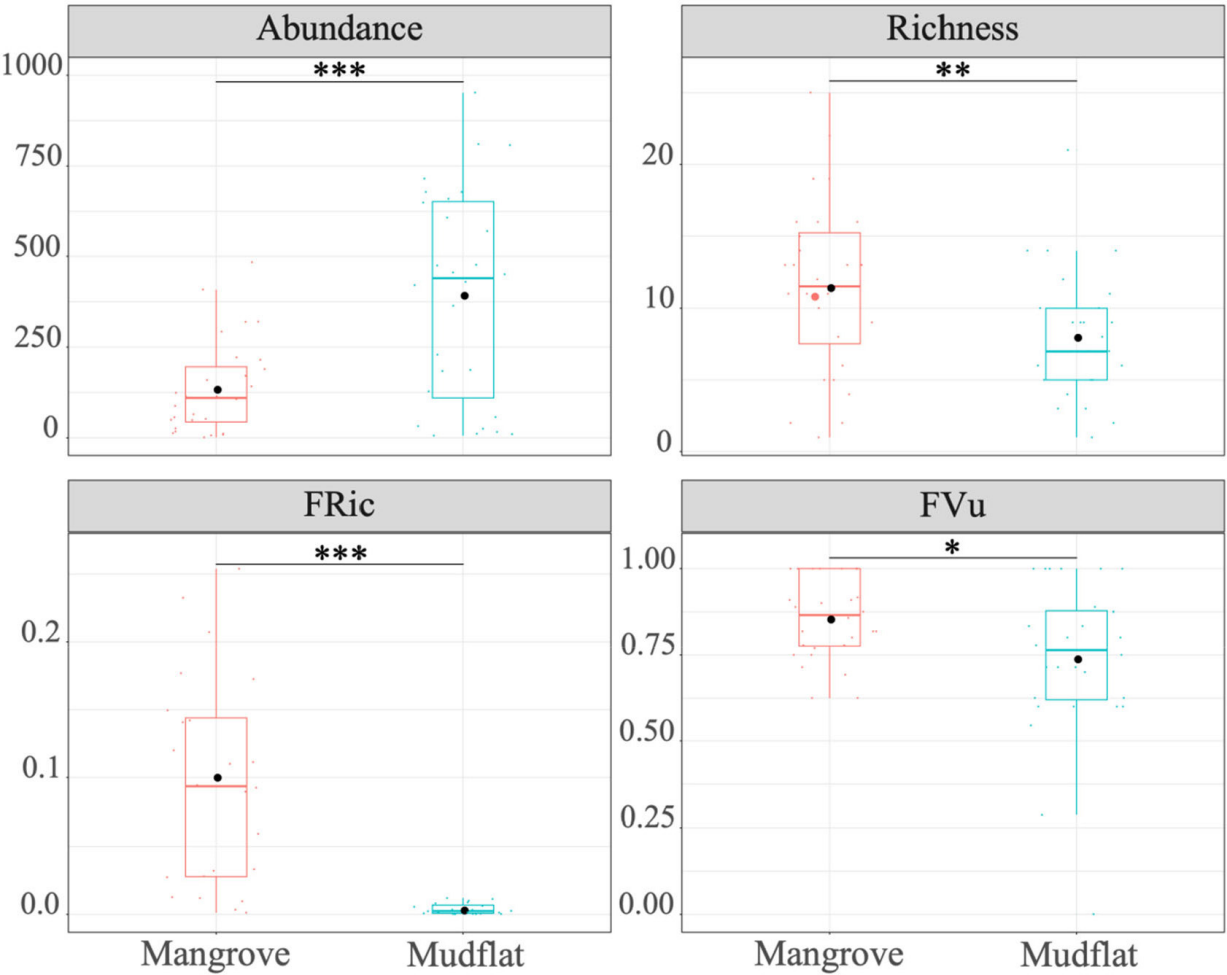
Results of Wilcoxon test of abundance, richness and functional diversity of mangrove molluscs between unvegetated mudflat and mangrove forest. The error bars represent the standard error (SE). The black dots represent the mean values. ‘***’ indicates very strong evidence (*p* < .001). ‘**’ indicates strong evidence (*p* < .01). ‘*’ indicates moderate evidence (*p* < .05). FRic, functional richness; FVu, functional vulnerability.

As shown in Figure 2, the abundance of molluscs in unvegetated mudflats was significantly higher than this in mangroves. However, species richness and functional diversity (FRic, FVu) of molluscs in unvegetated mudflats were significantly lower than those in mangrove forest. The comparison of CWM values of single trait between habitats showed that there was no significant difference in functional traits between mangroves and unvegetated mudflats (Table S2). In addition to differences in length, both unvegetated mudflats and mangrove forests were dominated by non‐climbing, free‐living, detrital and infaunal molluscs (Table S2).

### 3.2 Relative importance of biotic and abiotic drivers of mangrove mollusc biodiversity

**FIGURE 3:**
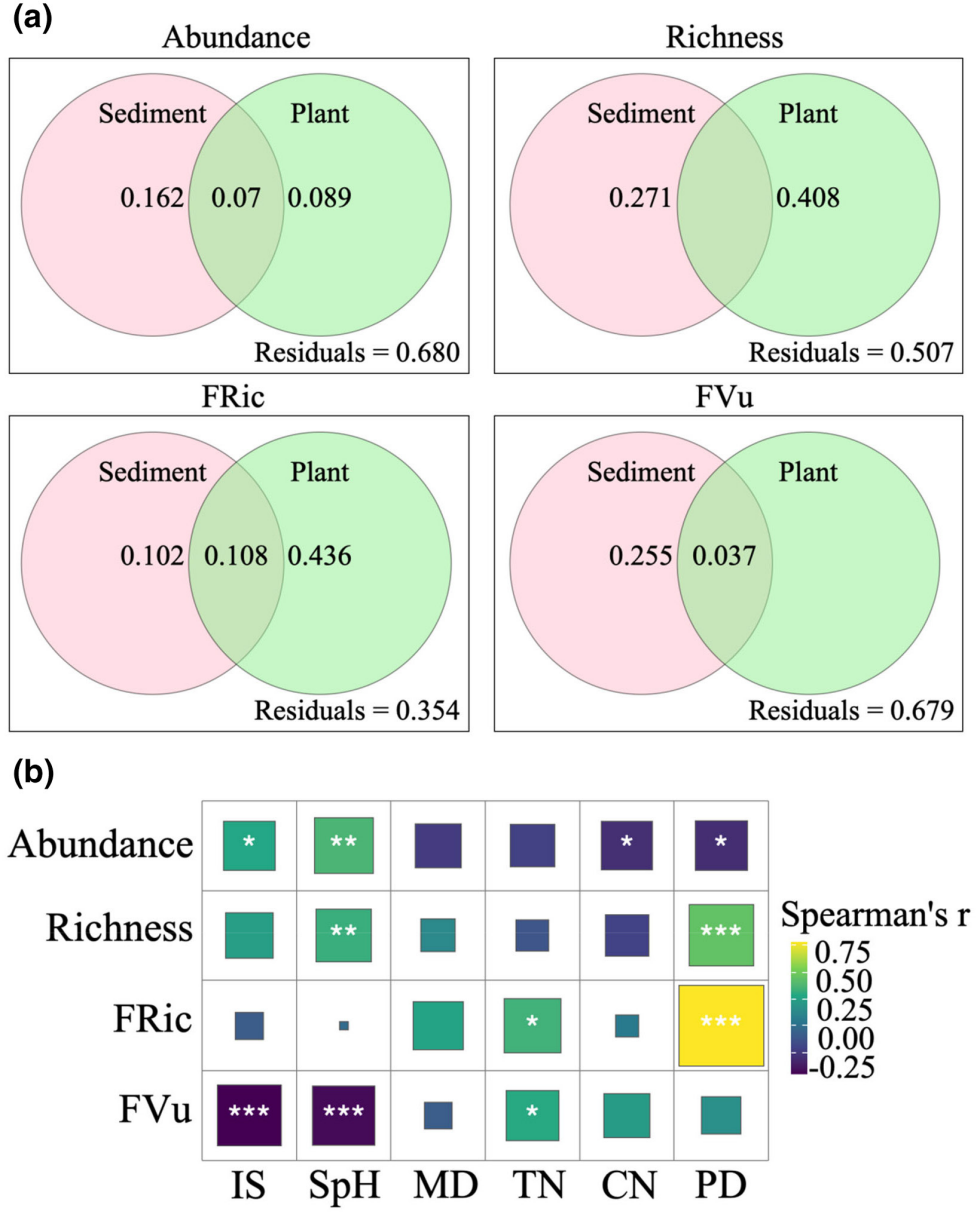
(a) The relative importance of abiotic and biological factors on mollusc communities detected by redundancy analysis and variation partition analysis (VPA). The Venn plot displays the percentage of total explained variation for each unique part. (b) Spearman correlation analysis of biodiversity and environmental factors. ‘***’ indicates very strong evidence (*p* < .001). ‘**’ indicates strong evidence (*p* < .01). ‘*’ indicates moderate evidence (*p* < .05). CN, the ratio of total organic carbon and total nitrogen in sediment; FRic, functional richness; FVu, functional vulnerability; IS, interstitial water salinity; MD, median diameter; PD, density of trees; SpH, pH of sediment; TN, sediment total nitrogen.

Six environmental variables (IS, SpH, MD, TN, CN and PD) are selected to assess the relative importance of biotic and abiotic factors for mangrove molluscs through Spearman rank correlation test (Figure S2 and Figure 3b).

VPA (Figure 3a) showed that sediment characteristics have a greater influence on abundance (16.2%) and FVu (25.5%). Abundance was significantly positively correlated with IS and SpH and negatively correlated with CN, while FVu was significantly negatively correlated with IS and SpH and positively correlated with TN (Figure 3b). Species richness and FRic of molluscs were mainly affected by vegetation structure, with an independent contribution of 40.8% and 43.6% to the explained variance, respectively (Figure 3a). According to correlation analysis, PD was significantly positively correlated with both species richness and FRic (Figure 3b).

### 3.3 The optimal curve for the proportion of unvegetated mudflat area to total area and mangrove mollusc biodiversity

**FIGURE 4:**
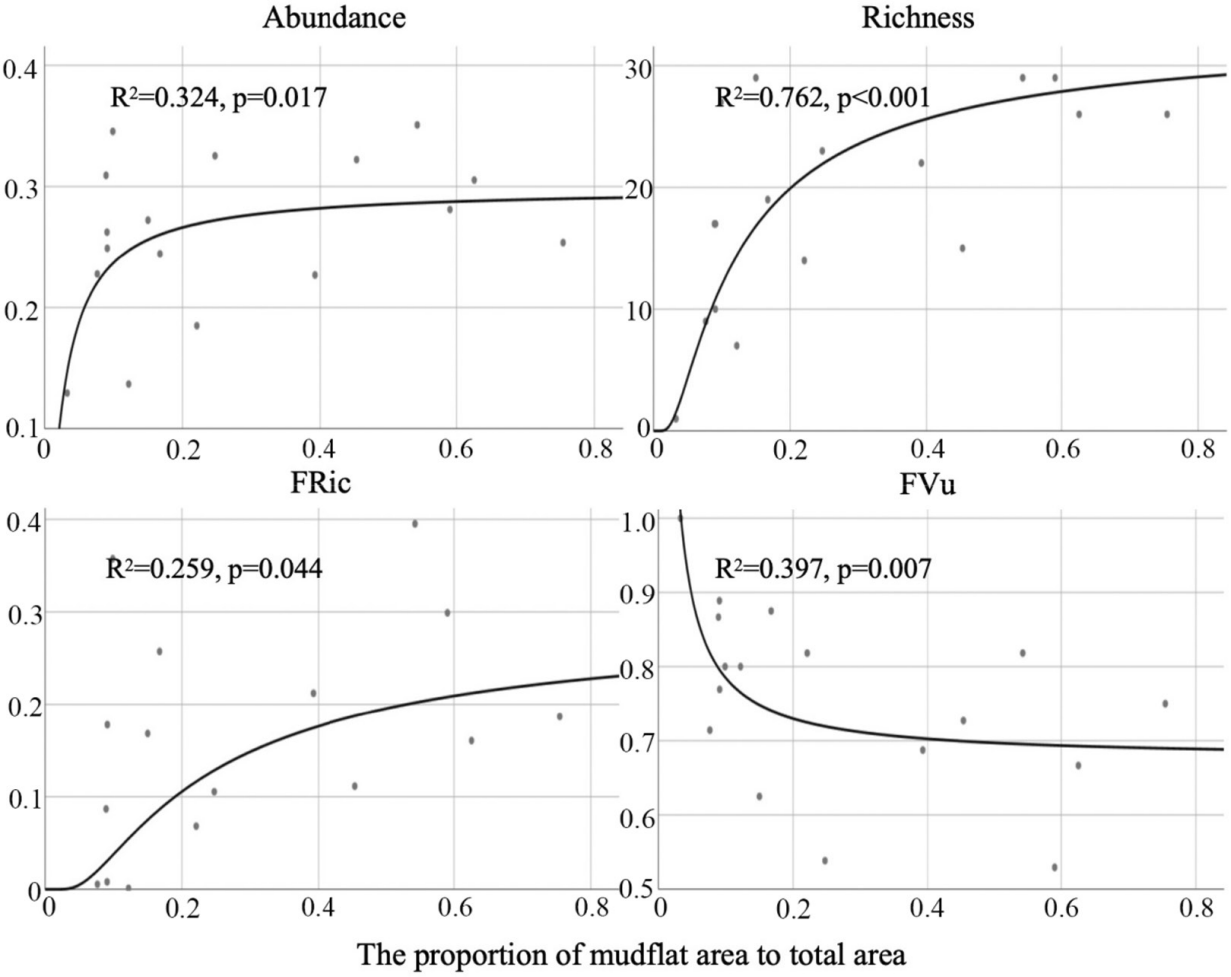
Visualization of the optimal curve for the proportion of unvegetated mudflat area to total area and mangrove mollusc biodiversity. The grey dots represent the sampling points. The solid black line is the fitted curve. The fitted regression was significant at *p* < .05 but was non‐significant at *p* > .05. The black dots represent the sampling points. FRic, functional richness; FVu, functional vulnerability.

According to Table 2, mollusc abundance, species richness and function richness (FRic) were all the S function showing the best fit, while functional vulnerability (FVu) was the reciprocal function showing the best fit. With the increase of the proportion of unvegetated mudflat area, abundance, richness and FRic increased and FVu decreased (Figure 4). When the proportion of unvegetated mudflat area reached 20%, all indexes showed a slow increase or decrease trend and gradually became stable (Figure 4).

**TABLE 2:** Results of fitting a range of mathematical functions to the ratio of mudflat area to total area [*x*] and mollusc biodiversity [*f*(*x*)].

Biodiversity	Mathematical model	The model summary					Parameter estimate			
		*R* ^2^	*F*	df1	df2	Sig.	Constant	b1	b2	b3
Abundance	Linear function	0.13	2.245	1	15	0.155	2.318	1.026		
	Logarithmic function	0.197	3.674	1	15	0.075	3.127	0.319		
	Reciprocal function	0.27	5.535	1	15	0.033	2.962	−0.048		
	Quadratic function	0.189	1.629	2	14	0.231	2.005	4.049	−4.138	
	Cubic function	0.191	1.026	3	13	0.413	2.11	2.538	0.61	−4.029
	Compound function	0.14	2.446	1	15	0.139	2.2	1.606		
	Power function	0.22	4.242	1	15	0.057	3.212	0.15		
	S function	0.324	7.188	1	15	0.017	1.095	−0.023		
	Increasing function	0.14	2.446	1	15	0.139	0.789	0.474		
	Exponential function	0.14	2.446	1	15	0.139	2.2	0.474		
Richness	Linear function	0.37	8.816	1	15	0.01	12.503	22.632		
	Logarithmic function	0.478	13.763	1	15	0.002	29.486	6.514		
	Reciprocal function	0.509	15.562	1	15	0.001	25.256	−0.857		
	Quadratic function	0.408	4.824	2	14	0.025	9.215	54.38	−43.46	
	Cubic function	0.446	3.487	3	13	0.047	3.926	130.269	−281.883	202.352
	Compound function	0.261	5.292	1	15	0.036	9.269	6.238		
	Power function	0.472	13.398	1	15	0.002	42.872	0.623		
	S function	0.762	47.91	1	15	0	3.496	−0.101		
	Increasing function	0.261	5.292	1	15	0.036	2.227	1.831		
	Exponential function	0.261	5.292	1	15	0.036	9.269	1.831		
FRic	Linear function	0.167	2.816	1	14	0.116	0.099	0.215		
	Logarithmic function	0.18	3.077	1	14	0.101	0.258	0.063		
	Reciprocal function	0.175	2.974	1	14	0.107	0.236	−0.012		
	Quadratic function	0.187	1.499	2	13	0.26	0.061	0.559	−0.46	
	Cubic function	0.211	1.068	3	12	0.399	0.142	−0.489	2.64	−2.535
	Compound function	0.205	3.616	1	14	0.078	0.032	27.242		
	Power function	0.246	4.565	1	14	0.051	0.401	1.018		
	S function	0.259	4.9	1	14	0.044	−1.223	−0.205		
	Increasing function	0.205	3.616	1	14	0.078	−3.442	3.305		
	Exponential function	0.205	3.616	1	14	0.078	0.032	3.305		
FVu	Linear function	0.207	3.912	1	15	0.067	0.825	−0.244		
	Logarithmic function	0.331	7.436	1	15	0.016	0.629	−0.078		
	Reciprocal function	0.397	9.886	1	15	0.007	0.675	0.011		
	Quadratic function	0.336	3.537	2	14	0.057	0.913	−1.089	1.157	
	Cubic function	0.363	2.469	3	13	0.108	0.978	−2.02	4.08	−2.481
	Compound function	0.191	3.533	1	15	0.08	0.818	0.724		
	Power function	0.299	6.393	1	15	0.023	0.632	−0.102		
	S function	0.338	7.648	1	15	0.014	−0.396	0.014		
	Increasing function	0.191	3.533	1	15	0.08	−0.201	−0.323		
	Exponential function	0.191	3.533	1	15	0.08	0.818	−0.323		

Abbreviations: FRic, functional richness; FVu, functional vulnerability.

## 4 DISCUSSION

### 4.1 Differences in habitat use by molluscs in mangrove wetlands

Mangrove wetland in Hainan Island is an important habitat for molluscs. In this study, 88 species of molluscs were recorded, which were more species than Li et al. (2022). Our study confirmed marked differences in mollusc biodiversity between mangrove forests and unvegetated mudflats. Vegetated habitats support relatively higher taxonomic (species richness) and functional diversity (FRic) than unvegetated habitats (Figure 2). The studies by Nozarpour et al. (2024) and Leung and Cheung (2017) had similar results. And richness and FRic were more influenced by vegetation structure (Figure 3a). The complexity of vegetation structure was considered to be one of the factors affecting its diversity (Leung, 2015). For example, mangrove roots provide complex microhabitats that facilitated sediment deposition and enhance food availability, thereby reducing the top‐down influence of predators (Corte et al., 2021; Hajializadeh et al., 2020). In addition, the density of mangrove plants was highly significantly positively correlated with the density of *Ovassiminea brevicula* in the mangrove forest of *Avicennia alba* in Thailand, possibly because the canopy provided microhabitats conducive to the growth of molluscs (Suzuki et al., 2002), which was consistent with the results of our study (Figure 3b). It had been found that high vegetation density leads to low underground animal community diversity, while medium and low vegetation density promoted underforest animal diversity (Zhang et al., 2003). The ground within mangrove forests was covered with a large amount of leaf litter, providing a substantial surface area for the attachment of macrobenthos (Nozarpour et al., 2024). However, research had shown that there was no significant difference in functional diversity between mangrove vegetation habitat and unvegetated habitat, which indicated that functional diversity may exhibit little or no dependence on vegetation structure (Nozarpour et al., 2023). Macrobenthic organisms possess the capacity to perceive and utilize habitats commensurate with their body size (Robson et al., 2005). Ecologically speaking, body size mirrors disturbance, movement of organic matter and biological interactions within a community (De Roos et al., 2003; Veríssimo et al., 2012). Our study showed that molluscs in unvegetated mudflats were larger in body size than those in mangrove forests (Table S2). This may be due to the fact that dense root structures could provide shelter from predators for small‐sized species and limit the movement and feeding of large‐sized species (Leung, 2015). In both unvegetated mudflats and vegetated habitats, the proportion of detritivores was higher (Table S2). Detritivores played an important role in nutrient cycling and energy transfer within mangrove ecosystems (Lee & Silliman, 2006). Many mangrove systems supported large communities of leaf litter that were consumed by species such as sesarmid and ocypodid crabs, which, in turn, controlled the fate and distribution of mangrove detritus, affecting detritivore communities (Lopez & Levinton, 2011).

Previous studies of mangrove macrobenthos had reported less abundance within mangrove forests than in adjacent habitats such as seagrass meadows and open sand/mudflats (Alfaro, 2006; Checon et al., 2017; Corte et al., 2021; Leung, 2015). Our results confirmed these findings, showing that the abundance within the mangrove forest was significantly less than that of the adjacent mudflats (Figure 2). Molluscs, especially gastropods, can reach high abundance and biomass in mangrove ecosystems (including mangrove forests and unvegetated mudflats) and occupy different levels in the food web, thus exerting a substantial influence on energy flow and material export to other ecosystems (Ebadzadeh et al., 2024). Sediment properties (mainly particle size, salinity, total organic carbon, total nitrogen and pH) were the main drivers of macroinvertebrate community assembly (Abdullah Al et al., 2022; Alsaffar et al., 2020; Hajializadeh et al., 2020; Wang et al., 2019), which supported our findings (Figure 3). Total organic carbon and total nitrogen serve as surrogate indicators of nutritional resources, fulfilling the energetic requirements of benthic organisms (Kröncke et al., 2003). The leaf litter within mangrove habitats had a high C/N ratio (Bouillon et al., 2002; Nerot et al., 2009; Schwamborn et al., 2002). Our study found a significant negative correlation between the C/N ratio and the abundance of molluscs (Figure 3b). Sediments with a lower C/N ratio generally indicated higher nitrogen content, which allowed animal communities to accumulate sufficient energy in a shorter time, thus enhancing reproductive rates (Lee, 2008). Fluctuations in salinity and pH affect the availability, accumulation and transport of heavy metals in some mangrove plants (Al‐Asif et al., 2023), which in turn affect benthos. Ma et al. (2018) found that on the western coast of Hainan Island, the lower salinity due to freshwater inflow in Maniao Bay and Huachang Bay led to a decrease in both the number of mollusc species and their diversity. Changes in plant communities can also indirectly affect benthic ecological processes by altering environmental factors (Chen, Wang, Zhang, et al., 2020). The degradation of mangrove plant communities has become a global issue (Duke et al., 2007; Wang et al., 2020). During changes in mangrove plant communities, environmental factors such as hydrological conditions were altered, affecting associated marine organisms (Chen, Wang, et al., 2021). On one hand, mangroves often lead to habitat sediment acidification and salinization, and changes in plant communities often alter sediment acidification and salinization (Xiao et al., 2020). On the other hand, the mangrove wetlands on the western coast of Hainan Island have numerous aquaculture ponds and residential areas (Fu, Zhang, et al., 2021). The discharge of aquaculture wastewater and domestic sewage lowers water salinity and acidity, affecting the dispersion and colonization of benthic animals (Chen, Wang, Liu, et al., 2020).

Quantifying biodiversity vulnerability (i.e. the extent to which biodiversity and related functions are likely to change in the face of multiple threats) is essential to rationalize ecosystem management and conservation actions (Auber et al., 2022). The functional vulnerability of molluscs in mangrove habitats was significantly higher than that in unvegetated mudflats (Figure 2), suggesting that mangrove habitats may be less resistant to specific disturbances in the future, such as typhoons and extreme low temperatures and so on. Notably, even within biodiverse systems like temperate and tropical regions, the most abundant marine mammal communities exhibited heightened vulnerability (Parravicini et al., 2014), underscoring the notion that species richness and diversity ought not always to be construed as a protective shield but rather as a potential buffer against vulnerability.

In general, although the taxonomic diversity (species richness) and functional diversity (FRic) of molluscs in mangrove forest were higher than those in unvegetated mudflat regions, the unvegetated mudflat regions supported more abundance and had lower functional vulnerability. Therefore, the relative importance of mangrove forest and mudflat regions should be balanced in the process of mangrove wetland protection and restoration.

### 4.2 Implications of the proportion of mangrove forest and unvegetated mudflat area for mangrove wetland conservation and restoration

In recent years, the area of mangroves in the world has undergone substantial reduction, primarily attributed to land reclamation and aquaculture practices (Friess et al., 2019). Concurrently, urbanization, pollution and extreme climatic events have contributed to extensive mangrove degradation or mortality (Polidoro et al., 2010). Ambitious large‐scale mangrove restoration targets have been set around the world (Friess et al., 2022). For example, Indonesia planned to restore 600,000 ha of mangroves by 2024 (Sasmito et al., 2023). In 2020, China introduced the Special Action Plan for Mangrove Protection and Restoration (2020–2025), which aimed to create and restore 18,800 ha of mangroves by 2025 (Choi et al., 2022; Jia et al., 2018). In 2022, Mozambique announced a plan to plant 50–100 million mangrove seedlings across 185,000 ha of land (Friess et al., 2022). The dramatic decline of mangrove area around the world was initially curtailed, with the annual decline rate of global mangrove area decreasing from 1%–2% to 0.16%–0.39% (Goldberg et al., 2020; Hamilton & Casey, 2016; Richards & Friess, 2016). Afforestation of mudflats represents the important approach to mangrove restoration (Agoramoorthy, 2012). Due to a lack of suitable coastal areas for restoration, efforts had been shifted to marginal spaces with less land use rights. The main reason the Philippines was keen on mudflats afforestation was that mudflats were open public land, avoiding issues related to land ownership (Primavera et al., 2014). This was also the case in China. From 2000 to 2019, China's mangrove area increased by about 8000 ha, with over 90% attributed to mudflats afforestation (Wang et al., 2021). However, although these areas had available space, the natural environment was biophysically unsuitable for the establishment and growth of mangroves (Gatt et al., 2022). Reports indicated that despite decades of efforts and funding, the success rate of mangrove restoration remained generally poor (Dale et al., 2014; Hashim et al., 2010; Kairo et al., 2001; Lewis, 2005; Primavera & Esteban, 2008). For example, from 1989 to 1995, West Bengal in India afforested 9050 hm^2^, but only 138 hm^2^ successfully became mangrove forests (Wang et al., 2021). The survival rate of artificially planted mangroves in the Philippines was only 10%–20% (Primavera & Esteban, 2008). An analysis of recent mangrove restoration projects in Sri Lanka found that out of 23 planting sites, 9 had a survival rate of 0%, and only 3 sites had a survival rate exceeding 50% (Kodikara et al., 2017). In Guangxi, China, the survival rate of mangrove afforestation from 2008 to 2015 was only 26.6% (Fan & Mo, 2018), and by 2019, the mangrove survival rate in Zhejiang Province, China, was approximately 22.4% (Chen et al., 2019).

Many factors behind low survival rates stem from the fact that incentive‐based restoration (especially area‐based or seedling‐number‐based targets) often provided poor incentives for restoration practitioners (Friess et al., 2022). However, despite the efficacy of these objectives and regulations in promoting mangrove expansion, certain problems and deficiencies persist. For example, the optimal allocation of mangrove and unvegetated mudflat area was not explored based on field investigation to balance the relative benefits of mangrove expansion and mudflat protection. Studies have shown a rapid decline in mudflats around the world over the past few decades (Murray et al., 2019). In addition to direct losses caused by coastal development, mangrove expansion may also contribute to the decline (Murray et al., 2019). Unvegetated mudflats are important habitats for benthic organisms and birds and serve as natural corridors for mangroves to cope with rising sea levels. Planting mangroves on mudflats was considered habitat conversion rather than habitat restoration (Ragavan et al., 2021). The transition from mudflat systems to mangrove forests is likely to entail shifts in hydrological conditions and biomes, including reduced flow rates (Lee & Shih, 2004), substitution of shorebirds and waterfowl by tree‐dwelling egrets, reduced production of benthic relative to mangrove litter and alterations in benthic dominance from polychaetes and amphipods to crabs (Huang et al., 2012). Unvegetated mudflats support unique biomes, especially molluscs, which constitute a primary food source for shorebirds. Thus, some studies advocate utilizing historical baselines to guide mangrove reforestation efforts, preferably preserving both mangroves and unvegetated mudflats within the same area (Liu & Ma, 2024). Local managers and stakeholders often resort to the active removal of mangrove seedlings or the felling of mangrove stumps to impede mangrove expansion due to conflicting needs between mangroves and shorebirds in intertidal afforestation, yet such actions are not consistently supported by local policies (Choi et al., 2022). Consequently, during the initial phases of mangrove afforestation, a certain proportion of mudflat area should be conserved to maintain the connectivity and integrity of each geomorphic unit within the mangrove wetland, alongside augmenting mangrove forest area. Yang et al. (2021) had concluded that the appropriate ratio of mudflats area to mangrove area in mangrove ecosystem was 3.5662 or 3.9785 by exploring the richness and abundance of shorebirds. This means that the optimal mudflat area accounts for 78.1% or 79.9% of the total area. Notably, our study, focusing on benthic perspectives, advocates reserving at least 20% of the mudflat area for fauna in mangrove wetland restoration initiatives (Figure 4). This may be due to the fact that shorebirds have a larger home range than molluscs, so they require a larger area. Global data on long‐term trends in mudflat ecosystems showed that the rate or scale of maintaining mudflat extent was insufficient to counteract the ongoing loss of these ecosystems (Murray et al., 2019). Given the exacerbation of climate change and anthropogenic disturbances, extensive and substantial conservation actions, restoration, supplementation and remediation are needed worldwide to address the multiple factors causing ecosystem loss. Additionally, it is imperative to integrate diversity considerations into policy formulation and management, particularly in decisions encompassing broader temporal and spatial scales. There is an urgent need for science‐based mangrove and mudflat ecosystem protection and restoration efforts to halt and potentially reverse severe wetland degradation. Our findings furnish a scientific underpinning for such endeavours.

## 5 CONCLUSION

Both mangroves and unvegetated mudflats are equally important in protecting and supporting biodiversity during mangrove ecological restoration. Based on a survey of molluscs in mangrove wetlands in Hainan Island, our study concludes the following: first, although species richness, functional richness and functional vulnerability of molluscs in the unvegetated mudflat regions were significantly lower than those in the mangrove forest, their abundance was relatively higher. Furthermore, sediment characteristics predominantly influenced mollusc abundance and functional vulnerability, whereas vegetation structure exerted a greater impact on species richness and functional richness. Most importantly, to more effectively protect mangrove wetland biodiversity, we recommend expanding mangrove areas while maintaining at least 20% of unvegetated mudflat areas. Hence, we advocate for heightened attention to the significance of unvegetated mudflats alongside the expansion of mangrove areas during the mangrove ecological restoration process. This dual approach will not only safeguard various species' habitats but also sustain the overall functionality and stability of the ecosystem. Our study is the first to propose the proportion of mangrove forests and unvegetated mudflats based on benthic biodiversity, furnishing scientific underpinning and decision‐making insights to facilitate the execution of large‐scale mangrove ecosystem conservation and restoration endeavours, thereby advancing the cause of mangrove ecosystem protection.

## AUTHOR CONTRIBUTIONS

**Yufeng Lin:** Conceptualization (lead); data curation (lead); formal analysis (lead); investigation (lead); methodology (lead); visualization (lead); writing – original draft (lead); writing – review and editing (lead). **Zifeng Luo:** Investigation (equal); writing – review and editing (equal). **Xuan Gu:** Conceptualization (equal); formal analysis (equal); investigation (equal); methodology (equal); visualization (equal); writing – original draft (equal); writing – review and editing (equal). **Yijuan Deng:** Investigation (equal); writing – review and editing (equal). **Pingping Guo:** Investigation (equal); writing – review and editing (equal). **Guogui Chen:** Conceptualization (equal); formal analysis (equal); methodology (equal); writing – review and editing (equal). **Wenqing Wang:** Conceptualization (equal); data curation (equal); formal analysis (equal); funding acquisition (lead); methodology (equal); project administration (lead); supervision (equal); writing – original draft (equal); writing – review and editing (equal). **Mao Wang:** Conceptualization (equal); data curation (equal); formal analysis (equal); funding acquisition (equal); methodology (equal); project administration (equal); supervision (lead); writing – original draft (equal); writing – review and editing (equal).

## CONFLICT OF INTEREST STATEMENT

The authors declare that the research was conducted in the absence of any commercial or financial relationships that could be construed as a potential conflict of interest.

## Supporting information


Data S1

## Data Availability

The raw data in this article are available from the Zenodo repository (https://doi.org/10.5281/zenodo.11634494).
