# Supplementary material for: Optimizing mangrove afforestation: Mollusc biodiversity comparisons reveal optimal mudflat–mangrove area ratio
Source: Ecol Evol. 2024 Sep 22;14(9):e70330. doi: 10.1002/ece3.70330 (PMC11416864; doi:10.1002/ece3.70330)
Supplement: Supplementary file 1 — Data S1 [file ECE3-14-e70330-s001.docx]

Supplementary material for

**“Optimizing mangrove afforestation: mollusc biodiversity comparisons reveal optimal mudflat-mangrove area ratio”**


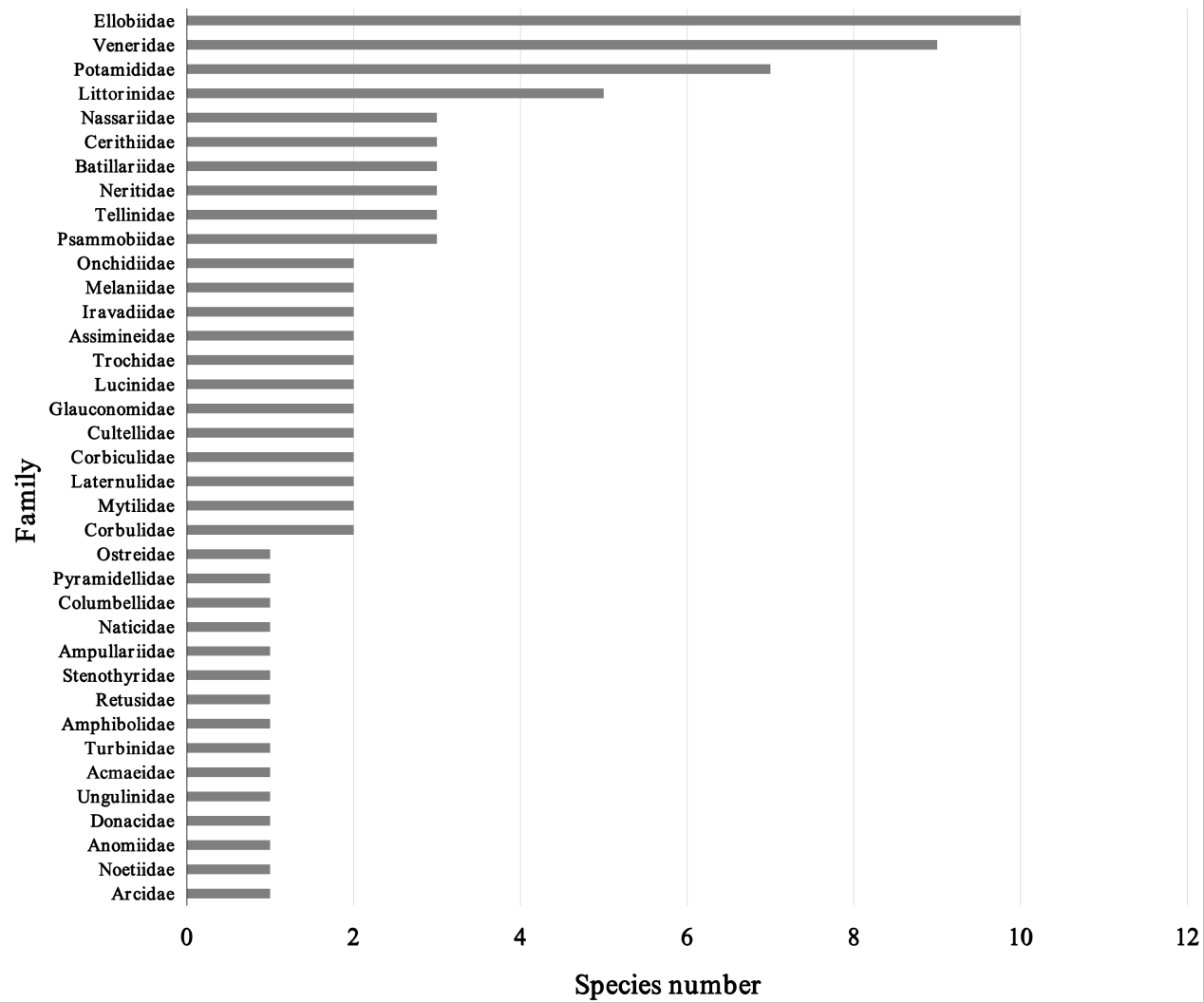


**Figure A.1.** Number of species of molluscs by family in Hainan Island

**Table A.2.** Differences in the single-trait community-weighted mean trait values (CWMs) between mangrove and unvegetated mudflat

| **Biological trait** | **Trait category** | **CWM** | | |
| --- | --- | --- | --- | --- |
|  |  | **Mangrove** | **Mudflat** | **p** |
| Climb | Never | 0.51(±0.33) | 0.94(±0.17) | >0.05 |
|  | Occasional | 0.36(±0.35) | 0.06(±0.17) | >0.05 |
|  | More | 0.1(±0.18) | 0(±0.01) | >0.05 |
|  | Permanent | 0.03(±0.11) | 0(±0.02) | >0.05 |
| Feed | Herbivorous | 0.05(±0.12) | 0.09(±0.22) | >0.05 |
|  | Filter feeder | 0.21(±0.34) | 0.18(±0.33) | >0.05 |
|  | Detritivous | 0.72(±0.37) | 0.72(±0.38) | >0.05 |
|  | Scavenger | 0(±0.01) | 0.01(±0.03) | >0.05 |
|  | Carnivorous | 0(±0) | 0(±0.02) | >0.05 |
|  | Omnivorous | 0.01(±0.06) | 0(±0) | >0.05 |
| Habitat | Sessile | 0(±0) | 0(±0) | >0.05 |
|  | Attachment | 0.02(±0.11) | 0(±0.02) | >0.05 |
|  | Free | 0.98(±0.11) | 1(±0.02) | >0.05 |
| Length | 1-5 mm | 0.01(±0.01) | 0.01(±0.03) | >0.05 |
|  | 5-10 mm | 0.39(±0.35) | 0.07(±0.17) | >0.05 |
|  | 10-20 mm | 0.1(±0.19) | 0.13(±0.26) | >0.05 |
|  | 20-30 mm | 0.2(±0.22) | 0.48(±0.35) | >0.05 |
|  | 30-40 mm | 0.13(±0.16) | 0.22(±0.26) | >0.05 |
|  | 40-50 mm | 0(±0.02) | 0(±0.01) | >0.05 |
|  | >50mm | 0.17(±0.3) | 0.09(±0.24) | >0.05 |
| Vertical | Leaf | 0.01(±0.02) | 0(±0) | >0.05 |
|  | Branch | 0.09(±0.19) | 0.01(±0.02) | >0.05 |
|  | Epifauna | 0.7(±0.34) | 0.8(±0.34) | >0.05 |
|  | Infauna | 0.19(±0.31) | 0.19(±0.33) | >0.05 |


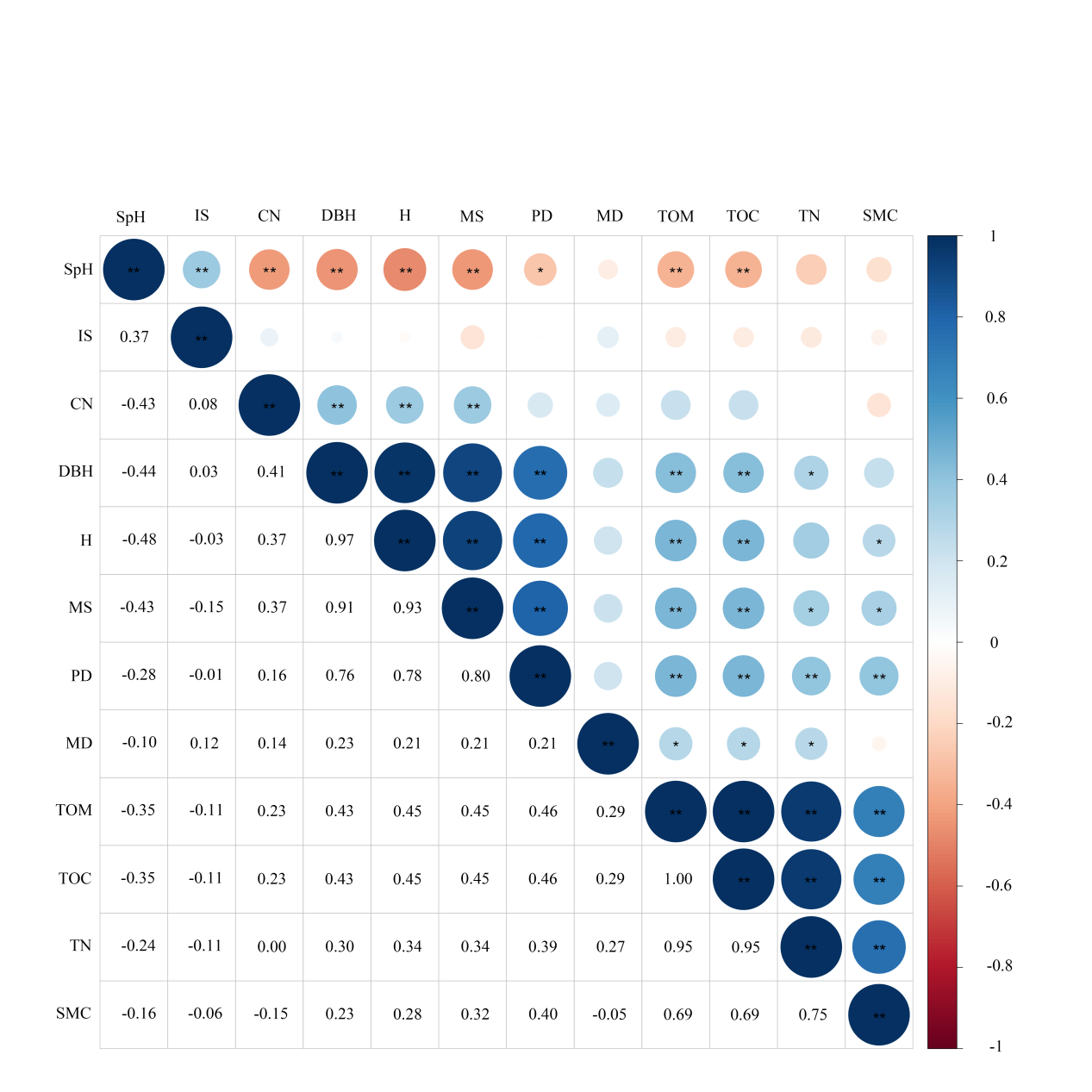


**Figure A.2.** Spearman correlation analysis of biotic and abiotic factors. *p < 0.05; **p < 0.01. No signal indicates little or no evidence (p > 0.05). SpH: pH of sediment; IS: interstitial water salinity; CN: the ratio of total organic carbon and total nitrogen in sediment; DBH: diameter at breast height; H: height of tree; MS: number of mangrove species; PD: density of trees; MD: median diameter; TOM: total organic matter of sediment; TOC: sediment total organic carbon; TN: sediment total nitrogen; SMC: water content of sediment.
